# Quantitative visual tests in primary open-angle glaucoma patients according to three different lights with different color-rendering index

**DOI:** 10.1186/s12886-021-02005-2

**Published:** 2021-05-28

**Authors:** Sang Woo Kim, YoungWook Go, Sang-Ook Kang, Chang Kyu Lee

**Affiliations:** 1 Department of Ophthalmology, Ulsan University Hospital, University of Ulsan College of Medicine, 877 Bangeojinsunhwando-ro, Dong-gu, 44033 Ulsan, South Korea; 2 GL Vision Co., Ltd, Seo-myeon, Republic of Korea; 3 Department of Advanced Materials Chemistry, Korea University, Seoul, South Korea; 4 Biomedical Research Center, Ulsan University Hospital, University of Ulsan College of Medicine, Ulsan, South Korea

**Keywords:** glaucoma, quantitative visual test, quantum dot LED, color rendering index

## Abstract

**Purpose:**

To compare quantitative visual tests, such as visual acuity, contrast sensitivity, and color vision tests in patients with primary open-angle glaucoma (POAG) patients according to three different light systems with different color-rendering index (CRI).

**Methods:**

This was a cross-sectional study of 36 eyes in 36 patients with POAG. Three different light systems consisting of a 3-band fluorescent lamp (CRI 80), a white LED (CRI 75), and a quantum dot LED (CRI > 95) were used. All lights had the same illuminance of 230 lx to exclude illuminance effects. The visual testing included best-corrected visual acuity (BCVA) using an ETDRS chart, a CSV-1000E contrast test, and a color test performed by the Farnsworth Munsell 100-hue test.

**Results:**

There was no significant difference in BCVA (*p* = 0.86). There were no significant differences in the detail contrast tests according to the three light systems (*p* = 0.95, *p* = 0.94, *p* = 0.94, respectively, *p* = 0.64). There was significant difference between the three light systems in color test (*p* = 0.042). The color test scores with a quantum dot LED were significantly lower than those of the *white LED and 3-band fluorescent lamp (p = 0.03 and 0.047, respectively).*

**Conclusions:**

POAG patients did not show significant differences in visual acuity scores and contrast test scores, expressed as black and white symbols, according to the different light systems. However, POAG patients tested under a quantum dot LED (CRI > 95) could distinguish color differences better than in the other light systems.

## Introduction

Glaucoma is a chronic, progressive heterogeneous group of diseases characterized by cupping of the optic nerve head and irreversible visual impairment. [1] It has become the most frequent cause of irreversible blindness worldwide[2, 3]. Patients with glaucoma may suffer from the inconvenience of using anti-glaucoma drugs, economic concerns for the cost of anti-glaucoma treatment, and obstacles faced with the visual impairment of glaucoma. Also, glaucoma impacts individuals in several ways, including a reduced ability to perform self-care activities[4], decreased steroacuity[5], being prone to traffic accidents,[6] depression,[7] and falls[8]. Thus, the quality of life (QoL) is strongly influenced by glaucoma. Specifically, impairment in visual acuity, contrast acuity, and color discrimination due to glaucoma negatively impacts physical and mental health and is a global concern.

Visual function also may be affected by artificial light sources because artificial light is common in the daily life of most people who consume around 2650 billion MWh/year, almost 19 % of the worldwide electricity production.[9] Traditionally, incandescent lights have been used as artificial light sources. However, as of the first of September 2016, no more incandescent lights were available in Europe for domestic lighting due to environmental problems and solid-state lighting based on light-emitting diodes (LEDs) has become the next-generation light source. Compared to conventional general illumination solutions, such as incandescent bulbs and compact fluorescent lamps, white LEDs have several advantages, including high luminous efficacy (> 70 lm W^− 1^), long lifetime (> 25,000 h), dimmability, and fast response times. [10] Moreover, several values are used to measure and judge the performance and color quality of artificial light sources, including LEDs. These are the color-rendering index (CRI), the correlated color temperature (CCT), and visual energy efficiency (luminous efficacy of radiation, LER), among others.[11, 12].

Thus, the purpose of this study was to evaluate three different kinds of light systems affect on quantitative visual tests in glaucoma patients and determine if the visual function in glaucoma patients was affected by the CRI degree used in the typical and traditional color quality metrics of artificial light systems.

## Methods

### Subjects

 This study adhered to the tenets of the Declaration of Helsinki and Ulsan University Hospital Institutional Review Board approval was obtained (UUH 2021-01-010). We obtained informed consents from all subjects. The study was a cross-sectional study and all subjects were enrolled between June 2017 and May 2019.

The subjects in the POAG group were recruited from the Department of Ophthalmology at Ulsan University Hospital, South Korea. The inclusion criteria for the POAG group were (1) a cup-to-disc ratio ≥ 0.5 or > 0.2 difference in the cup to disc ratio of each eye, (2) a defect in the retinal nerve fiber layer (consistent with a glaucomatous change in the optic nerve) on either a fundus photograph taken using red-free light or an optical coherence tomography image, (3) evidence of glaucomatous visual field loss using a Humphrey Field Analyzer (with the Swedish Interactive Threshold Algorithm-Standard 30 − 2 or 24 − 2 program), and (4) open angle was assessed by van Herick method first. If anterior chamber depth was shallower than grade 3 (PAC = 1/4 to 1/2CT), gonioscopic examination was used to confirm open angle. All POAG patients were diagnosed by one glaucoma specialist (C.K.L).

The exclusion criteria were as follow: best-corrected visual acuity < 0.1 on a decimal scale or > 1.0 on the logarithm of the minimum angle of resolution scale; the presence of uveitis or other retinal diseases; severe cataract (> 3 nuclear opalescence classified by LOCS III)[13]; myopia of ≥ − 6 D; age > 65 or < 18 and a history of cerebrovascular disease, ocular trauma.

The sample size was calculated by using of our pilot study results. Sample number of pilot was five and within group standard deviation (SD) was 81.2. One-sided alpha level was set as 0.05, the power was 80 %, and dropout rate was 10 %. Finally, the study population of each group was calculated 36.

**Fig. 1:**
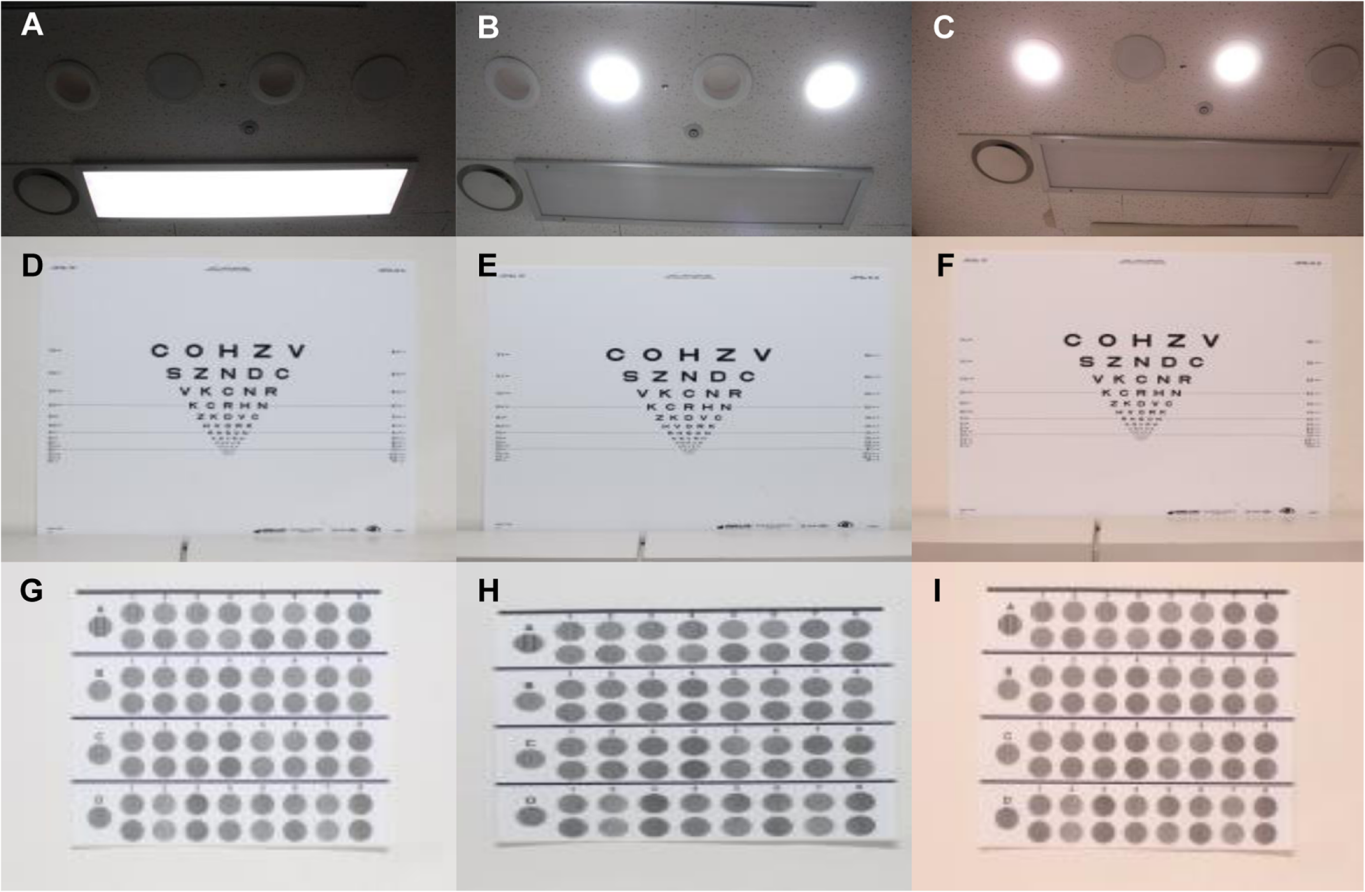
The ETDRS chart and CSV-1000E chart under each of three different light systems. (**A**)3-band fluorescent lamp (CRI: 80). (**B**) White LED (CRI: 75). (**C**) Quantum dot LED (CRI > 95). (**D**, **G**) ETDRS and CSV-1000E charts under the 3-band fluorescent lamp. (**E**, **H**) ETDRS and CSV-100E charts under the white LED. (**F**, **I**) ETDRS and CSV-1000E charts under the quantum dot LED

All quantitative visual tests were performed in our light research lab. All tests were administered to all patients in turn through group 1,2 and 3 order and BCVA test was first performed, followed by CSV and color tests in the same order for all patients. There were three different light systemes in this study; a 3-band fluorescent lamp that had a CRI of 80 was classified as Group 1, a general white LED that had a CRI of 75 and was classified as Group 2, and a quantum dot LED that had a CRI as 95 and was classified as Group 3. The light sources were suspended from the ceiling of our lab. In the white and quantum dot LED, an attenuator was fitted to control the illumination. All quantitative visual tests were conducted after controlling the illuminance from each source at 230 lx by using digital light meter (TES-1330 A, TES, Taiwan) because all visual acuity and contrast test were essentially needed under same illuminance [14] (Fig. 1).

### Best-Corrected Visual Acuity (BCVA)

BCVA was checked by ETDRS charts that were used for a test distance range of 3 m. One eye of each patient was tested and the eye to be tested was chosen by tossing a coin. There was a 5-minute break after each test under each light system to rule out fatigue effects and interference from the previous light system. This was commonly done by counting the number of letters read correctly on the entire chart and converting this to an acuity score by means of a simple formula that placed an L or N value on the letter, where L was the difference in acuity between adjacent lines and N was the number of letters per line. Thus, in a chart with five letters per line and a 0.1 LogMAR progression from line to line, each correct letter is worth 0.1/5 = 0.02LogMAR[15].

### Contrast Sensitivity Test (CSV)

Contrast sensitivity testing was done with a CSV-1000E (Vector Vision, Dayton, OH, USA)[16] The test distance was 2.5 m and one eye of each patient was tested and the eye to be tested was chosen by tossing a coin. There was a 5-minute break time after each test under each light system to rule out fatigue effects and interference from the previous light system. The CSV-1000E provides for four rows of sine-wave gratings with spatial frequencies of 3, 6, 12, and 18 cycles/degree (CPD). The CSV results are presented as log values according to a table provided by the manufacturer.

### Color test

Color testing was done by the Farnsworth-Munsell(F-M) 100 hue test (VeriVide, Enderby, United Kingdom)[17]. This test was the 85 colored- cap version. The caps are arranged in four boxes, each containing a fixed anchor cap at each end. There were 22 caps in box 1 and 21 caps in each of the remaining three boxes. Standard administration procedures were followed whereby, for each tray, the intermediate caps were removed from the tray and placed in a random arrangement while the participant looked away. The participant is then asked to view and place the intermediate caps in the correct order in the tray between the two fixed caps, with as little difference in hue between the neighboring caps as possible. The tray was completed in a different order by each participant. The order in which the participant placed the caps was recorded by the experimenter. The tests were conducted under the same illumination with different light systems and one eye of each patient was tested. There was a 10-minute break after each test under every light system to rule out fatigue effects and interference from the previous light system. Standard scoring procedures were followed. Error scores for each tray position were calculated based on the differences between the chosen cap and the two neighboring caps, generating a baseline score of 2 for each cap when in perfect order. Error scores for caps at the end of each tray were calculated using the neighboring cap in the same tray and the first cap of the next tray so that all caps were considered on a continuum around a color circle. The total error score (TES) was measured and computed, in which a higher number of misplacements is reflected by larger TES values. Also, age-matched normal persons were administered the same procedure for comparing results to the POAG patients.

### Statistical analysis

One-way ANOVA were used to analyze the data and a p-value of < 0.05 was considered statistically significant. if values were statistically significant, subsequent post hoc multiple comparison using Duncan revealed specific group difference. All statistical analyses were performed using Statistical Package for the Social Sciences (SPSS) for Windows (version 21.0, SPSS, Inc., Chicago, IL, USA).

## Results

Thirty six eyes of 36 POAG patients were included in this study. The age range of the patients was 15 − 64 years (mean age, 46.7 ± 14.2 years). The ratio between men and women was 61.1/38.9 %. The MD of visual field test in this group was − 10.5 ± 9.0 decibels (dB)(range, -2.00 to -26.78 dB) and the mean PSD in this group was 7.24 ± 4.7 dB (range, 1.75 to 15.08 dB) (Table 1).

**Table 1:** Baseline characteristics of the patients (20 eyes of 20 patients)

	Values		
Age, year (mean ± SD)	46.7 ± 14.2		
Sex, men/women (% )	61.1/38.9		
Laterality (OD/OS)(%)	38.9/61.1		
HVF MD (mean ± SD) (dB) PSD (mean ± SD) (dB)	-10.5 ± 9.0 7.24 ± 4.7		
HVF: Humphrey visual field, MD: mean deviation, PSD: pattern standard deviation, dB: decibel			

There was no significant difference in BCVAs in the three groups (*p* = 0.86) (Table 2). There was no significant difference in the detail contrast test (A, B, C, D) in the three groups (*p* = 0.95, *p* = 0.94, *p* = 0.94, *p* = 0. 64, respectively) (Table 3).

**Table 2:** BCVA values between the three groups by ETDRS (LogMAR)

	Mean	SD	P-value*
Group 1	0.25	0.16	0.867
Group 2	0.27	0.16	
Group 3	0.25	0.17	
* Kruskal-Wallis analysis; BCVA: best-corrected visual acuity; ETDRS: Early Treatment Diabetic Retinopathy Study; Group 1: 3-band fluorescent lamp, Group 2: white LED, Group 3: quantum dot LED; SD: standard deviation			

**Table 3:** Contrast test Values between the three groups by CSV-1000E (Log value)

	Groups	Mean	SD	P-value*
Contrast A	Group 1	1.59	0.27	0.95
	Group 2	1.59	0.24	
	Group 3	1.61	0.25	
Contrast B	Group 1	1.53	0.25	0.94
	Group 2	1.51	0.37	
	Group 3	1.49	0.33	
Contrast C	Group 1	1.06	0.30	0.94
	Group 2	1.03	0.32	
	Group 3	1.01	0.36	
Contrast D	Group 1	0.66	0.35	0.64
	Group 2	0.56	0.36	
	Group 3	0.58	0.38	

* Kruskal-Wallis analysis; Group 1: 3-band fluorescent lamp, Group 2: white LED, Group 3: quantum dot LED; SD: standard deviation

**Fig. 2:**
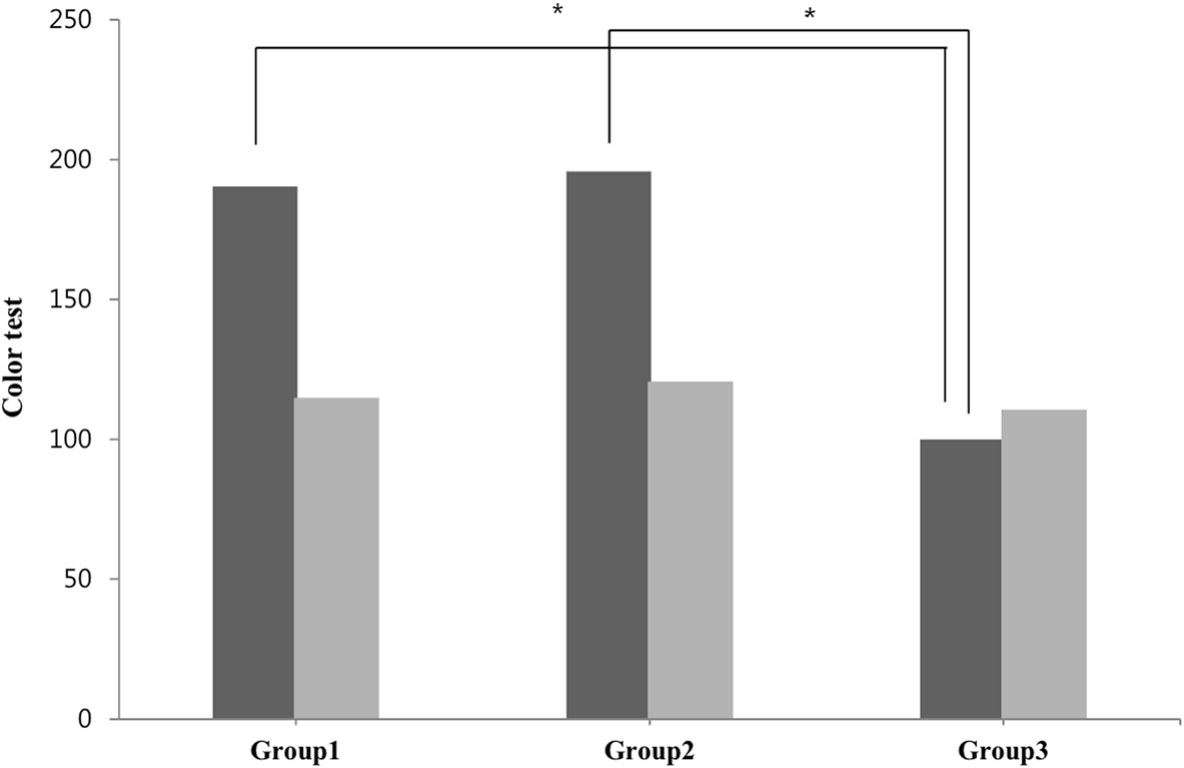
Relationship between color tests and groups (light systems). Black bar shows results of glaucoma patients and gray bar shows result of normal subjects that were age-matched to glaucoma patients. There were significant differences between group 1 and group 3, and group 2 and 3 in the TES of the color test in glaucoma patients(asterisk means significant difference) (*p* = 0.047, *p* = 0.03, respectively).There was no significant difference between groups in normal subjects. There was no significant difference between group 1 and group 2 (*p* > 0.05). Group 1: 3-band fluorescent lamp (CRI: 80), Group 2: White LED (CRI: 75), Group 3: Quantum dot LED (CRI > 95)

**Fig. 3:**
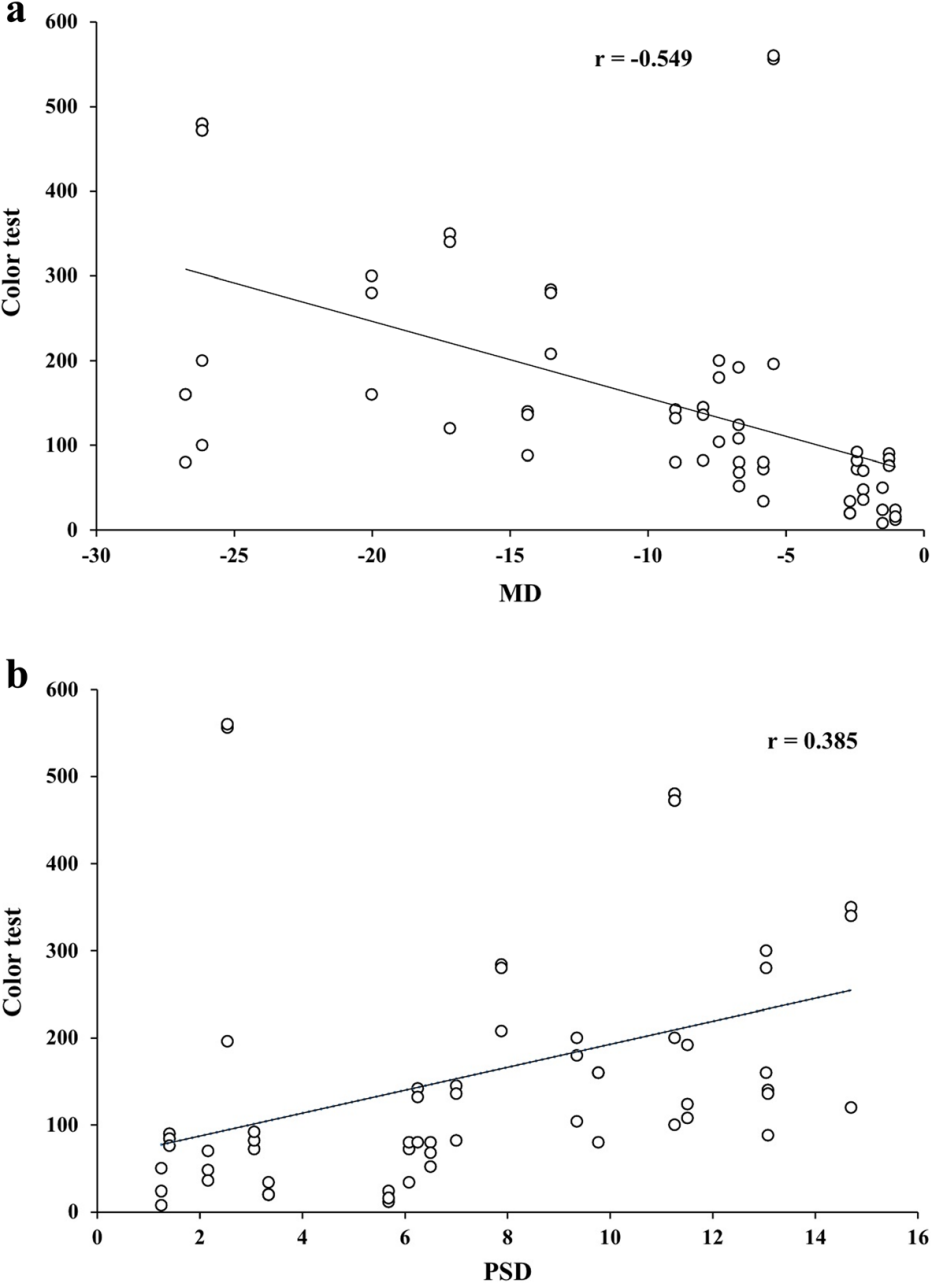
Relationship between the color test, MD, and PSD. (**A**)There was a significant negative relationship between the MD and TES of the color test (r = -0.549, *p* = 0.001). (**B**) There was a significant positive relationship between the PSD and TES of the color test (*r* = 0.385, *p* = 0.002)

The mean TES was significantly difference in between groups (*p* = 0.042)(Table 4) and the quantum dot LED group had significantly lower TES than other groups (Table 4; Fig. 2) Also, there were no significant differences of the TES according to three different light systems between non-glaucoma subjects age-matched to the study subjects (p > 0.05) (Fig. 2). The mean deviation, which is one of the Humphrey visual field indices, was significantly negatively correlated with the TES of the color test (*p* = 0.001)(Fig. 3A) and the pattern standard deviation, which is another Humphrey visual field index, was significantly positively correlated with the TES of color testing (*p* = 0.002)(Fig. 3B).

**Table 4:** Color test values between the three groups by the F-M 100 hue test.

	Mean	SD	P-value*
Group 1	190.4	169.1	0.042
Group 2	195.8	167.1	
Group 3	118.5	60.0	

* One-way ANOVA analysis, F-M: Farnsworth-Munsell; Group 1: 3-band fluorescent lamp, Group 2: general LED, Group 3: quantum dot LED, SD: standard deviation

## Discussion

This study showed that there was no difference in the ability of glaucoma patients to distinguish items in the visual acuity and contrast tests under different lighting systems, although the glaucoma patients, especially the POAG patients, had trouble distinguishing color depending on the quality of light in the different light systems. Also, according to our previous study, there was no difference in the ability of normal subjects to distinguish color under the same light system used to in this study[13]. The results of this study indicated that glaucoma patients had no difficulty distinguishing black and white under different types of light sources or different light quality if the different light systems had the same illuminance. However, the ability of glaucoma patients to distinguish color was significantly different according to the CRI. The CRI is one of the major and traditional performance metrics and constant values used to judge color quality and is well known as a color metric used to reproduce good saturated colors of illuminated objects under white conditions[12]. A good white light source must render the real colors of the objects it illuminates. This feature of light sources has great importance for indoor lighting applications. Moreover, under low ambient lighting, such as outdoor lighting, Reynham et al. indicated that good color rendition helped to increase road safety by improving color contrast.[18]The CRI was developed by the Commission Internationale de l’Eclairage (CIE) in 1971[19]and updated to its current form in 1995[20]. The best CRI value is 100, whereas the worst is -100.[11] Accordingly, a light system with a high CRI indicates that the light source illuminates the object’s true color perfectly and provides the capability for accurate color discrimination. Therefore, we presumed that glaucoma patients might have some trouble with color discrimination under light systems with low CRI scores and easily distinguish color under a light system with a high CRI, independent of the type of light system if the illuminance was the same. This finding is very important for glaucoma patients because it is strongly associated with vision-related QoL in glaucoma patients. Many previous studies have reported that the QoL of glaucoma patients was lower than normal individuals because visual impairment significantly affects QoL. [21–23] Color discrimination is a type of visual function, such as visual acuity and the contrast test. Therefore, low color discrimination could affect QoL in glaucoma patients. It is well known that acquired color vision defects occur in glaucoma [24–27]because defects in the photoreceptors and optic nerve fibers distort color perception[28, 29] and the color vision defect may become worse when the glaucomatous visual defect is more severe. [21, 27] Thus, in this study, glaucoma patients with acquired color vision defects could have more difficulty distinguishing color influenced by the quality of the light system, as indicated by factors such as the CRI rather than the type of light system and this will be more severe in patients with more severe glaucoma (Fig. 3). The reason the CRI differs according to the light source is that an incandescent lamp has a continuous spectrum and a fluorescent lamp has a discrete line spectrum. An incandescent lamp has a higher CRI, however, incandescent lamps have surpassed them in terms of energy efficiency.[11] White LEDs usually have a low CRI (< 70) and a high correlated-color temperature (CCT > 6000 K)[30], which is another typical and classic performance metric and negatively correlated with the CRI. In contrast, quantum dot LEDs have other advantages. They exhibit fine spectral tuning, achieved by their size control and narrow-band emission[31]. Therefore, with optimized spectral designs, the real color of objects can be rendered properly while achieving a warm white shade and good spectral overlap with the sensitivity of the human eye.[11] Moreover, quantum dot LEDs with a high color CRI are graded as third-generation LED lights by LED-related scientists and engineers[12].

Moreover, many glaucoma patients use multiple antiglaucoma drugs and when they use these drugs, the bottle cap color is the most commonly used method for identifying the glaucoma medications.[32] Therefore, if glaucoma patients with acquired color vision defects have trouble in a low-CRI light system, they may confuse other drugs with anti-glaucoma drugs and lead to low adherence with glaucoma treatment and low treatment efficacy.

One of the limitations of this study was that the ETDRS and CSV-1000E tests were recommended to use retro-illumination as the illumination source rather than projection or using direct illumination because projection or direct illumination have a lack of sufficient luminance and contrast. However, in our study, the light systems were hung from the ceiling of our lab and we could only use direct illumination for the tests. However, the most important factor during all the tests was illuminance and we checked in front of the test chart and the F-M color unit and then controlled the attenuation of the two LEDs to match the illuminance of the 3-band fluorescent lamp (230 lx).

Also, the effect of the color metric degree on glaucoma target tissues and target cells, such as optic disc and retinal ganglion cells, must be evaluated by *in vitro* and *in vivo* tests in detail and step by step. Because, high-quality light systems, such as those with a high CRI, help glaucoma patients improve color discrimination though, light could be a risk factor in glaucoma or be a protector to glaucoma according to light character. [33–35].

In conclusion, glaucoma patients with deficiencies in color discrimination could be affected, not by the type of light system with the same illumination, but the quality of the light system, especially the CRI. Patients with more severe visual field defects had a lower ability to discriminate color. Thus, a more high-quality light system could increase the ability of color distinction and may increase the QoL in glaucoma patients because the relationship between ability of color distinction and QoL has been closed. In future studies, the safety of the main target organ or cells in glaucoma affected by different light systems and quality should be investigated according to several light quality indices, such as the CRI and CCT, by *in vivo* and *in vitro* testing.

## Data Availability

The datasets used and analysed during the current study are available from the corresponding author on reasonable request.
